# *Scaphium affine* Ethanol Extract Induces Anoikis by Regulating the EGFR/Akt Pathway in HCT116 Colorectal Cancer Cells

**DOI:** 10.3389/fonc.2021.621346

**Published:** 2021-05-20

**Authors:** Hye Won Kawk, Gun-He Nam, Myeong Jin Kim, Sang-Yong Kim, Young-Min Kim

**Affiliations:** ^1^ Department of Biological Science and Biotechnology, College of Life Science and Nano Technology, Hannam University, Daejeon, South Korea; ^2^ Department of Food Science and Bio Technology, Shinansan University, Ansan, South Korea

**Keywords:** *Scaphium affine*, HCT116 colorectal cancer cells, anoikis, EGFR/Akt pathway, xenograft model

## Abstract

*Scaphium affine* ethanol extracts (SAE) is a species that has been shown to contain various physiological effects; however, its anticancer effects have yet to be revealed. We qualitatively evaluated β-sitosterol in SAE through high-performance liquid chromatography (HPLC). The cytotoxicity in HCT116 and HT29 colorectal cancer cells and CCD841 normal colon cells was confirmed through WST-1 assays. Selective cytotoxicity was observed in colorectal cancer cells, with greater cytotoxicity demonstrated in the HCT116 cell line. As such, the HCT116 colorectal cell line was selected for subsequent experiments. After HCT116 cells were treated with SAE, it was confirmed that the apoptosis rate was increased in a SAE dose-dependent manner through Annexin V assay. SAE further showed dose-dependent suppression of invasion through invasion assays. Anoikis induction through the EGFR/Akt pathway in HCT116 colorectal cancer cells was confirmed by Western blotting. The tumor suppressive effects of SAE was assessed *in vivo* using a xenograft model of human HCT116 colorectal cancer cells. As a result, we confirmed that SAE decreased tumor size in a dose-dependent manner and that p-EGFR and cleaved-caspase 3 in tumors were also regulated in a dose-dependent manner. This study showed that SAE, by containing β-sitosterol with proven anticancer effects, induces anoikis through the EGFR/Akt pathway in HCT116 colorectal cancer cells both *in vitro* and *in vivo*.

## Introduction

Cancer is a disease caused by the random proliferation and metastasis of cells. Its incidence is high, and it is considered to be the leading cause of death in developed countries (1). The incidence of colorectal cancer, one of the most prevalent malignant tumors worldwide, is rapidly increasing due to industrialization and urbanization (2). The main risk factors for colorectal cancer include abnormal diets, such as substantial consumption of foods containing high animal fats and sugars, and excessive alcohol consumption. Smoking, long-term use of nonsteroidal anti-inflammatory drugs, colorectal diseases, metabolic syndrome, and heredity (only 5% of cases) are also potential causes of colorectal cancer (3, 4). Symptoms of colorectal cancer include alternating diarrhea and constipation as well as the appearance of mucus and blood in the feces. These symptoms can be accompanied by colon infarction, anemia, weight loss, and abdominal lumps (5, 6).

The treatment of colorectal cancer mainly involves surgical resection of the tumor, combined with anticancer drugs or radiation therapy (7). However, these interventions lead to adverse effects such as reduced immunity, normal cells toxicity, genetic damage, and hair loss (8). Accordingly, various studies have been conducted on new bioactive substances with anticancer effects that have been developed by considering the efficacy of harmless natural substances to reduce the adverse effects of chemotherapy and to selectively suppress the growth and proliferation of cancer cells through various mechanisms (9, 10).

*Scaphium affine*, used in this study, is the seed of *S. affine*, belonging to the family Sterculiaceae. *S. affine* has been proven to possess various effects such as ulcer-protective, antioxidant, and anti-inflammatory effects, but no anticancer effects have yet been proven (11–14). β-sitosterol, which has been found to be a physiologically active ingredient in many natural products, has been proven to have anticancer effects. Therefore, β-sitosterol-containing natural substances may have anticancer effects on colorectal cancer (15–17).

Adherent cells sometimes acquire migration, adhesion, and proliferation properties in an unsuitable environment. Most cells perceive this as an abnormal phenomenon, resulting in the loss in normal cell-matrix interactions, cessation in cell cycle progression, and eventually the induction of a specific type of apoptosis known as anoikis. However, when anoikis resistance occurs, cancer cells gain the ability to metastasize and invade (18–20).

Epidermal growth factor receptor (EGFR) belongs to the ErbB receptor family of tyrosine kinases. EGFR expression is associated with tumor differentiation, apoptosis, metastasis, and angiogenesis. It also plays a key role in the induction of anoikis (21, 22). EGFR functionally modulates caveolin-1, which is a major component of lipid rafts and caveolae, and regulates cellular behaviors such as cholesterol homeostasis, including anoikis. Src, a non-receptor tyrosine kinase, is an activating factor of EGFR and regulates cell proliferation and growth by promoting downstream signaling (23). Akt is directly involved in cell proliferation and growth through the influence of Src signaling. When EGFR, a key factor in anoikis induction, is suppressed, caveolin-1, src, and Akt are suppressed. Inactivation of Akt inhibits cell proliferation and growth, which consequently leads to anoikis induction *via* the intrinsic apoptosis pathway (24–28).

Herein, we performed a qualitative evaluation of β-sitosterol in *S. affine* ethanol extracts (SAE). We aimed to verify the EGFR/Akt pathway-mediated anoikis-inducing effects of SAE *in vitro* in HCT116 colorectal cancer cells. In addition, we investigated the effects of SAE on tumor suppression *in vivo* using an HCT116 human colorectal cancer xenograft model in Balb/c-nu mice, which are ideal hosts for rapid growth of tumor cell lines due to their lack of immunity.

## Materials and Methods

### Reagents

The SAE (CA01-060) used in this study was obtained from the Korea Plant Extract Bank at the Korea Research Institute of Bioscience and Biotechnology (Daejeon, Korea). A voucher specimen (PBC-131AS) was kept in the herbarium of the Korea Research Institute of Bioscience and Biotechnology. The plant (70 g) was dried in the shade and powdered and was added to 1L of ethyl alcohol 95.0% (GR grade, Daejung Chemical & Metals Co., Ltd), and extracted through 30 cycles (40KHz, 1500W, 15 min ultrasonication-120 min standing per cycle) at room temperature using an ultrasonic extractor (SDN-900H, SD-ultrasonic Co., Ltd). After filtration (Qualitative Filter No.2, Hyundai Micro Co., Ltd.), SAE (1.62 g) was obtained. Various concentrations of SAE were prepared by dissolving in dimethyl sulfoxide (DMSO; Samchun Co., Ltd., Korea) and were refrigerated at -4°C for use. β-sitosterol, an indicator component, was purchased from Sigma-Aldrich (Danvers, MA, USA) and was dissolved in methyl alcohol (Samchun Co., Ltd., Korea). Gefitinib (20 mM) was purchased from Abcam (Cambridge, UK), and LY294002 (20 mM) was purchased from Calbiochem (San Diego, CA, USA). Both inhibitors were diluted with DMSO. WST-1 solution was purchased from Dogen Bio (Seoul, Korea). Primary antibodies against t-EGFR (cat #2232), p-EGFR (cat #2234), t-Akt (cat #9272), p-Akt (cat #9271), p-Src (cat #2101), t-caveolin-1 (cat #3267), p-caveolin-1 (cat #3251), p53 (cat #9282), Bcl-2 (cat #2876), Bak (cat #6947), PARP (cat #9542), and β-actin (cat #4967) were purchased from Cell Signaling Technology (Danvers, MA, USA), whereas t-Src (cat #sc-8056) antibody was purchased from Santa Cruz Biotechnology (Dallas, TX, USA). Caspase 3 (cat #ab2302) antibodies were purchased from Abcam (Cambridge, UK). Among the secondary antibodies, anti-mouse IgG and HRP-linked antibody were purchased from Cell Signaling Technology, while goat anti-human IgG H&L (HRP) was purchased from Abcam. The Muse™ Annexin V and Dead Cell Assay kit was purchased from Luminx (Austin, TX, USA).

### Identification of Active Compounds of SAE With High-Performance Liquid Chromatography (HPLC)

SAE was diluted to 1 mg/mL using methyl alcohol and filtered. β-sitosterol was diluted to 0.01 mg/ml in the same manner and used for qualitative analysis. All materials were analyzed by injection into HPLC 2694 separation modules (Waters, USA) with separation flow rate of 1 mL/min through a SunFire™ C-18 column (4.6 × 250 mm, 5 μm, SunFire, Germany). In addition, the mobile phase of all materials was detected using the Dual Absorbance Detector 2487 (Waters) at 205 nm for 1 h with 5:95 water to acetonitrile.

### Cell Culture

HCT116 and HT29 colorectal cancer cells and CCD841 normal colon cells were obtained from the American Type Culture Collection (ATCC, Manassas, VA, USA). HCT116 and HT29 colorectal cancer cells were grown in RPMI medium (HyClone, UT, USA) containing 1% antibiotics (100 U/mL penicillin and 100 mg streptomycin/mL) and 10% fetal bovine serum (HyClone) at 37°C in a 5% CO_2_ atmosphere. CCD841 normal colon cells were grown in DMEM (HyClone) containing 1% antibiotics (100 U/mL penicillin and 100 mg/mL streptomycin) and 10% fetal bovine serum (HyClone) at 37°C in a 5% CO_2_ atmosphere. After washing in PBS (HyClone), the cells were suspended in trypsin-EDTA (HyClone) every 2 days.

### WST-1 Assay

Cells were seeded at 4.0 × 10^5^ cells/ml in a 24-well plate for 24 h and were incubated with SAE (50, 75, 100, 125 and 150 μg/mL) for 24 h. The inhibitors, gefitinib and LY294002, were pretreated for 60 – 120 min prior to treatment with SAE (100 μg/mL). The cells were incubated with 100 μl/mL WST-1 solution for 60 min. The optical densities of the solutions were then quantified at a 450 nm wavelength using a FLUOstar Omega system (BMG Labtech, Germany).

### Determination of Apoptosis by Annexin V Staining

Cells were seeded at 1.0 × 10^6^ cells/mL in a 6-well plate. After 24 h incubation, the cells were treated with various concentrations of SAE (75 and 125 μg/mL) for 24 h. The cells were resuspended in PBS. Then, 100 μL of Muse™ Annexin V and Dead cell reagent was added to 100 μL of the resuspended cells. After incubation for 20 min at room temperature, the dyed cells were analyzed in a Muse™ Cell Analyzer (EMD Millipore).

### Cell Invasion Assay

An SPL insert hanging 24-well plate (SPL Life Sciences, Korea) with a pore size of 8 μm was used. Corning Matrix (Corning, NY, USA) was applied to the insert. Cells were seeded at 4.0 × 10^5^ cells/mL with serum-free RPMI media in the insert. The bottom of the 24-well plate included RPMI media containing 1% antibiotics (100 U/mL penicillin and 100 mg streptomycin/mL) and 10% fetal bovine serum. SAE (75 and 125 μg/mL) was then added to the insert and incubated for 24 h, followed by 10% trichloroacetic acid for 1 h, and 0.25% crystal violet (Sigma-Aldrich) for 2 h. The dyed HCT116 colorectal cancer cells were observed using an inverted microscope (×100 magnification, Axiovert 100, Zeiss, Germany).

### Western Blotting

Cells were seeded at 1.0 × 10^6^ cells/mL in a 6-well plate. After 24 h incubation, the cells were treated with various concentrations of SAE (75 and 125 μg/mL) for 24 h. Inhibitors, gefitinib and LY294002, were pretreated for 60 – 120 min prior to treatment with SAE (100 μg/mL). After 24 h, the cells were rinsed with precooled PBS, and proteins were extracted using RIPA buffer (Radioimmunoprecipitation assay buffer; 50 mM Tris-HCl, pH 7.6, 150 mM NaCl, 1% Triton X-100, 1% sodium deoxycholate, 0.1% SDS, 2 mM EDTA; ForBioKorea, Korea) containing 1X Halt Protease and Phosphatase Inhibitor cocktail (Thermo Fisher, USA). The extracted protein was quantified to 20 µg by Bradford analysis using a Bio-Rad protein assay dye reagent concentrate (Bio-Rad, CA, USA). Proteins were separated by size using 8% or 12% acrylamide gels and then transferred to a nitrocellulose membrane (GE Healthcare Life Sciences, MA, USA). Blocking was performed using 5% bovine serum albumin (BSA; MP Biomedicals, CA, USA). Primary antibodies (t-EGFR, p-EGFR, t-caveolin, p-caveolin, t-Src, p-Src, t-Akt, p-Akt, p53, Bcl-2, Bak, caspase 3, PARP, and β-actin) reacted overnight at 4°C, while secondary antibodies (anti-mouse IgG, HRP-linked Antibody and Goat Anti-Human IgG H&L (HRP) Antibody) reacted for 120 min at 4°C. Western blotting was repeated at least 3 times for each experiment. SuperSignal™ West Femto Maximum Sensitivity Substrate (ThermoFisher, MA, USA) was used to react with the bound antibody substrate. Protein expression was confirmed using a UVITEC gel imaging system (Philekorea, Seoul, Korea). All western blotting images were quantitatively represented using ImageJ (National Institutes of Health, Washington, D.C., USA).

### *In Vivo* Xenograft Model

Male, 4-week-old Balb/c nu/nu mice were obtained from Envigo (Indiana, USA) and were housed in sterile, filter-topped cages. A total of 18 mice were randomly and equally divided into 3 group. The experiments were conducted in 3 groups [the negative control (N), SAE 75 mg/kg/day, and SAE 100 mg/kg/day group). The animals were provided with the appropriate accommodation, environment, food, water, and care for their health and to minimize distress. The mice were maintained under specifically controlled conditions (ambient temperature 23 ± 2°C, humidity 50 ± 5%, 12 h light/dark cycle). Body weight was measured once per week. For tumor induction, HCT116 human colorectal cancer cells (2.5 × 10^5^ cells/0.25 mL) were subcutaneously injected into the left flank of mice of all 3 groups (negative control, SAE 75 mg/kg/day, and SAE 100 mg/kg/day). Seven days after the injection of cells, the presence of tumor was confirmed. The animals were treated with SAE at 75 mg/kg/day or 100 mg/kg/day for another 14 days. The tumor size was measured using a digital caliper (Asimeto, Germany) at 7-day intervals, and the tumor volume was calculated using the following formula: V = 0.5 × (length × width × height). The animals were euthanized by CO_2_ asphyxiation followed by cervical dislocation, and the tumors were collected for histological analysis. The animal study was reviewed and approved by the Hannam University Animal Experimental Ethics Approval Committee (NHU2020-6; Daejeon, Korea).

### Immunohistochemistry

The tumors were fixed in 3.7% formaldehyde (10% neutral buffered formalin, Samchun Chemicals, Pyeongtaek, Korea) for 48 h, embedded in paraffin (Leica 39601006, Leica, Hessen, Germany) using a Tissue Processor (ASP300s, Leica), and sectioned into 3 μM thick slices using a rotary microtome (Leica RM2255, Leica). Tumor tissue sections were pre-warmed for 40 min at 60°C oven and were deparaffinized in ultraclear xylene (AvantiK, NJ, USA) for 3 rounds of 7 min each, 99.9% ethanol (GD Trade, Korea) for 2 rounds of 3 min each, 95% ethanol (GD Trade) for 2 rounds of 3 min each, 80% ethanol for 3 min, and 70% ethanol for 3 min using automatic H&E staining (Tissue-TeK, Prisma E2, Sakura, Japan). The tumor sections were treated with 3% H_2_O_2_ for 15 min, and non-specific binding blocking was performed using 5% BSA. The sections were then treated with a primary antibody (p-EGFR, cleaved-caspase 3) for 60 min at 4°C, and subsequently with a secondary antibody (HRP-conjugated anti-rabbit Ig) for 30 min. The sections were added to the DAB solution, incubated for 7 min, and rinsed with tap water for 10 min for DAB development. The sections were incubated with hematoxylin for 3 min, rinsed with tap water for 10 min, and dehydrated using an automated system (70%, 80%, 95%, 100% ethanol, xylene). All images were quantitatively represented using ImageJ.

### TUNEL Assay

Apoptosis was assessed using the TdT-mediated dUTP nick-end labeling (TUNEL) method. The tumors were fixed in 3.7% formaldehyde (10% neutral buffered formalin) for 48 h, embedded in paraffin, and sectioned into 3 μM thick slices. The paraffin was removed at 60°C for 40 min, and the tumor tissue sections were deparaffinized in ultraclear xylene for 3 rounds of 7 min each, 99.9% ethanol for 2 rounds of 3 min each, 95% ethanol for 2 rounds of 3 min each, 80% ethanol for 3 min, and 70% ethanol for 3 min using automatic H&E staining. Tumor tissue sections were processed using the ApopTag Peroxidase *in situ* Apoptosis Detection Kit (Vector Laboratories, USA). DAB solution was added and incubated for 7 min, and rinsed with tap water for 10 min for DAB development. Tumor tissue sections were incubated with Mayer hematoxylin for 3 min, rinsed with tap water for 10 min, and dehydrated using an automated system (70%, 80%, 95%, 100% ethanol, Xylene). All images were quantitatively represented using ImageJ.

### Statistical Analysis

All experiments were repeated at least 3 times. Statistical analyses were performed using the t-test (SPSS, Inc., Chicago, IL, USA). p < 0.05, p < 0.01, and p < 0.001 were considered to indicate a statistically significant difference. Error bars represent standard error.

## Results

### Qualitative Analysis of Bioactive Compounds (β-sitosterol) in SAE

**Figure 1:**
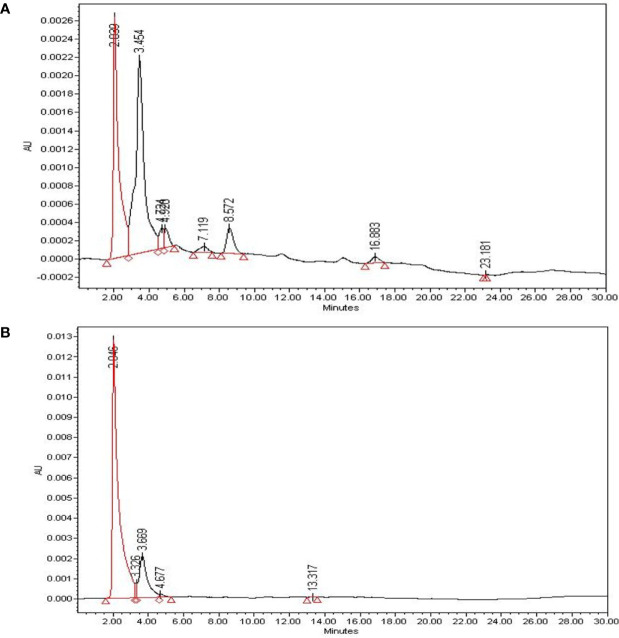
The identification of bioactive compounds in SAE. **(A)** HPLC profiles of SAE at 1 mg/mL (total extract). **(B)** HPLC profiles of standard β-sitosterol at 0.01 mg/mL (2.046 min). The X-axis represents retention time (min), and the Y-axis represents absorption units (AU). The detector was set at 205 nm.

β-sitosterol, a bioactive compound with proven anticancer effects, was qualitatively evaluated through HPLC of SAE. To identify β-sitosterol in SAE, we compared the retention time of the compound analyzed in SAE to that of β-sitosterol (**Figures 1A, B**). HPLC analysis of β-sitosterol in SAE and standard compound analysis showed a retention time of 2.039 min, confirming that β-sitosterol is present in SAE. By setting β-sitosterol as an indicator component, it (i.e. β-sitosterol) may act as a surrogate marker for anticancer effects of SAE.

### Cytotoxicity of SAE in HCT116 and HT29 Colorectal Cancer Cells by WST-1 Assays

**Figure 2:**
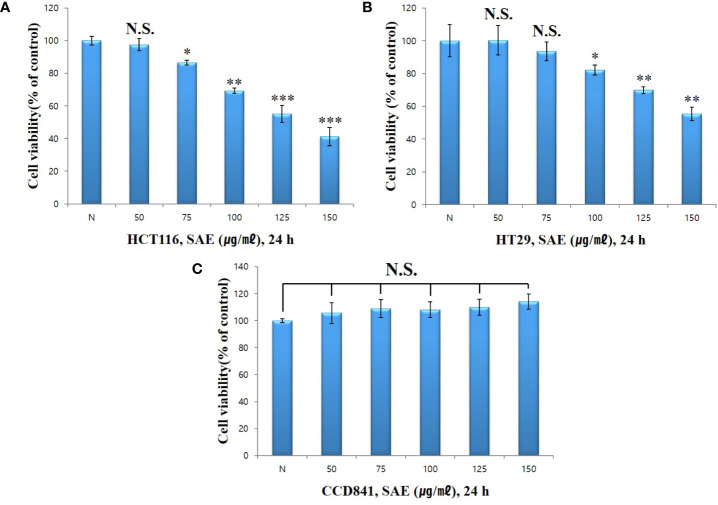
The effect of SAE on the viability of HCT116, HT29, and CCD841 cells. Cell viability was measured by WST-1 assays. **(A)** HCT116 cells were pretreated with SAE for 24 h. **(B)** HT29 cells were pretreated with SAE for 24 h. **(C)** CCD841 cells were pretreated with SAE for 24 h. Statistical analysis was performed using t-tests. *p < 0.05, **p < 0.01 and ***p < 0.001 compared to the controls. N.S., not significant (each experiment, n = 3). N., Negative control; 50., SAE 50 µg/mL; 75., SAE 75 µg/mL; 100., SAE 100 µg/mL; 125., SAE 125 µg/mL; 150., SAE 150 µg/mL.

To verify the cytotoxicity of SAE in HCT116 and HT29 colorectal cancer cells, both cell lines were treated with different concentrations of SAE(50, 75, 100, 125, and 150 µg/mL) for 24 h. Cytotoxicity was measured using WST-1 assays. HCT116 colorectal cancer cells showed % cell viability of 97.5%, 86.6%, 69.3%, 55.2%, and 41.2% at 50, 75, 100, 125, and 150 µg/mL, respectively (**Figure 2A**), whereas HT29 colorectal cancer cells showed % cell viability of 100.3%, 93.5%, 82.2%, 69.8%, and 55.4%, respectively (**Figure 2B**). These results indicated the dose-dependent cytotoxicity of SAE on HCT116 and HT29 colorectal cancer cells and that cytotoxicity was more effective on HCT116 colorectal cancer cells. In addition, when CCD841 normal colon cells were treated with SAE under the same conditions, no cytotoxicity was observed as % cell viability remained at 90% or more at all concentrations (**Figure 2C**). Therefore, the cytotoxicity of SAE was shown to be specific to HCT116 and HT29 colorectal cancer cells. Due to the greater cytotoxicity shown in HCT116 colorectal cancer cells, subsequent experiments were conducted on this cell line.

### Effect of SAE on Apoptosis Induction, and Invasion Inhibition in HCT116 Colorectal Cancer Cells

**Figure 3:**
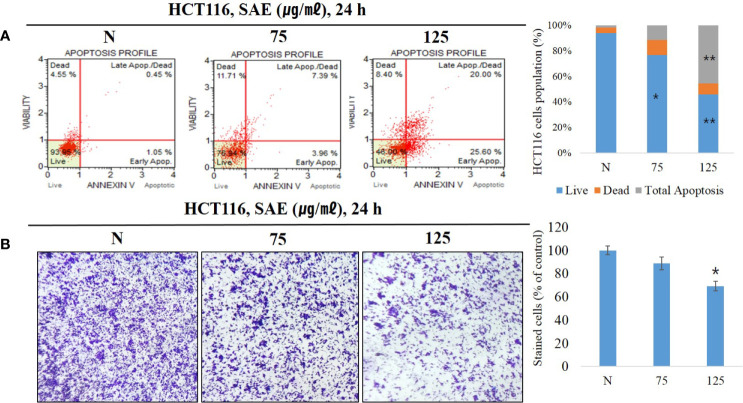
HCT116 cells were treated with SAE at different concentrations (75 and 125 µg/mL) for 24 h. **(A)** The apoptotic effects of SAE were evaluated using the Muse™ Annexin V assay. Data were analyzed by flow cytometry. **(B)** Cell invasion was measured using invasion assays. Statistical analyses were performed using t-tests; *P < 0.05, **P < 0.01 compared to N groups (each experiment, n= 3). N., Negative control; 75., SAE 75 µg/mL; 100., SAE 100 µg/mL.

Anoikis are a form of apoptosis caused by lack of intercellular interaction or loss of adhesion. To confirm that apoptosis was induced, we treated HCT116 colorectal cancer cells with 75 and 125 μg/mL SAE for 24 h, and phosphatidylserine (PS) expressed during apoptosis was stained with Annexin V. The staining ratio of PS was identified at each concentration of SAE using a flow cytometer to determine the ratio of apoptosis. The results showed that apoptosis was induced at 1.50% in the N group, 11.35% with 75 μg/mL SAE, and 45.60% with 125 μg/mL SAE. In addition, 75 ug/mL of low toxicity also involved apoptosis in induction by stimulation, but no significance was observed (**Figure 3A**). These results indicated that SAE induced apoptosis in HCT116 colorectal cancer cells in a dose-dependent manner. Anoikis resistance is a prerequisite for metastasis and invasion in cancer cells, and the induction of anoikis in an abnormal environment inhibits this behavior of cancer cells. A cell invasion assay was used to assess the inhibitory effects of SAE on HCT116 colorectal cancer cell invasion, and SAE was shown to suppress invasion in a dose-dependent manner (**Figure 3B**). These results highlighted that the anoikis-inducing effects of SAE associated with the invasion-inhibitory effect of SAE.

### Effects of SAE on Anoikis Signaling Protein Expression in HCT116 Colorectal Cancer Cells

**Figure 4:**
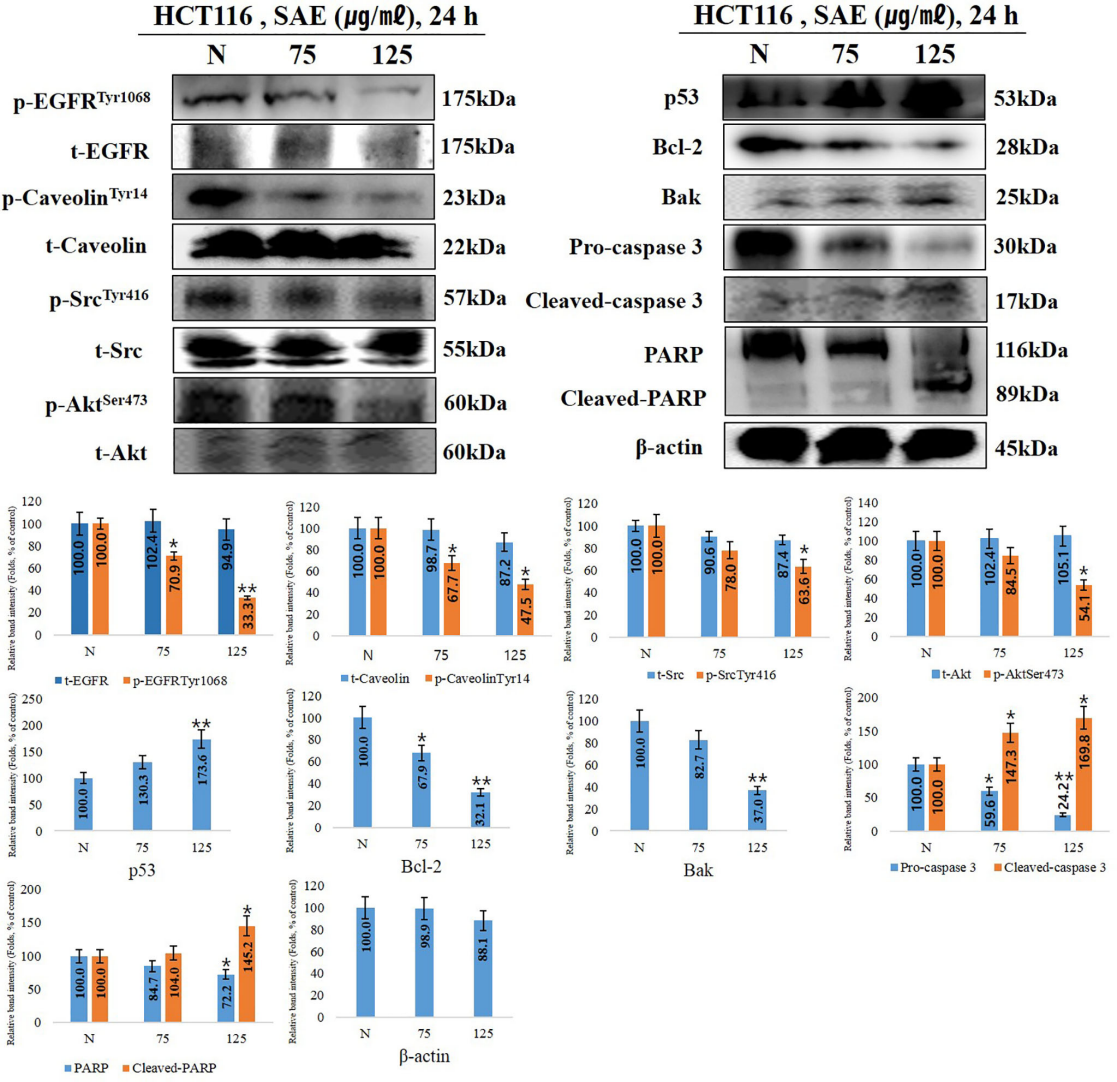
The effects of SAE on the expression of anoikis regulatory proteins. HCT116 cells were treated with the indicated concentrations of SAE for 24 h. The expressions of p-EGFR, p-caveolin, t-caveolin, p-Src, t-Src, p-Akt, p53, Bcl-2, Bak, PARP, and cleaved-PARP. Pro-caspase 3, cleaved-caspase 3, and β-actin were analyzed by Western blotting (each experiment, n = 3). All Western blotting images were quantitatively represented using ImageJ (National Institutes of Health). Statistical analyses were performed using t-tests; *P < 0.05, **P < 0.01 compared to N groups (each experiment, n= 3). The error bars represent the standard error. N., Negative control; 75., SAE 75 µg/mL; 100., SAE 100 µg/mL.

When anoikis occurs, the cell membrane receptor p-EGFR, a key factor in anoikis, is inhibited, thereby deactivating p-caveolin and p-Src. Accordingly, p-Akt, which regulates cell growth and proliferation, is deactivated, and tumor suppressor p53 is activated to induce the intrinsic apoptotic pathway. In this experiment, the trend of anoikis-inducing signaling proteins in HCT116 cells following 75 and 125 μg/mL SAE exposure for 24 h was examined. The results showed that SAE dose-dependently decreased p-EGFR and p-caveolin-1, which decreased p-Src. p-Akt showed a tendency to decrease, while p53 increased. Furthermore, SAE decreased Bcl-2, which maintains mitochondrial membrane potential, and increased Bak, which loss of mitochondrial membrane potential. Caspase 3 fragments PARP, which repairs DNA. And cleaved-caspase 3, an activated form of caspase 3, showed a tendency to increase, while pro-caspase 3 decreased in a dose-dependent manner. As the activity of caspase 3 increased, cleaved-PARP, the deactivated form of PARP, increased. The above changes were also observed at 75 ug/mL of low toxicity by stimulation. Overall, these results demonstrated that the concentration-dependent anoikis-inducing effects of SAE in HCT116 colorectal cancer cells proceeds by inducing the intrinsic apoptotic pathway through the regulation of anoikis-related signaling proteins such as EGFR, caveolin-1, Src, and Akt (**Figure 4**).

### Effects of EGFR/Akt Inhibitors on the Survival Rates of HCT116 Colorectal Cancer Cells the Expression of Anoikis Signaling Proteins

**Figure 5:**
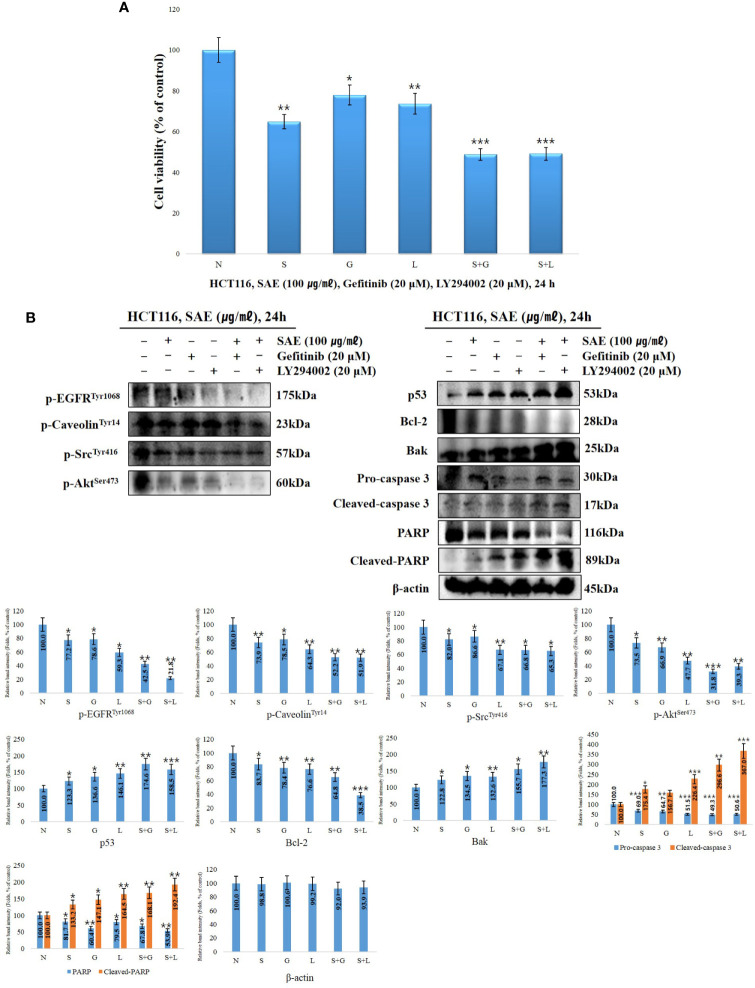
The effects EGFR/Akt inhibition on cell proliferation and signaling protein expression. HCT116 cells were treated with 20 μM gefitinib, 20 μM LY294002, and 100 μg/mL SAE for 24 h. **(A)** Cell viability was measured by WST-1 assays. Statistical analyses were performed using t-tests; *p < 0.05, **p < 0.01, ***p < 0.001. (each experiment, n = 3). N., Negative control; S., SAE; G., Gefitinib; L., LY294002. **(B)** The cells were treated with 20 μM gefitinib, 20 μM LY294002, and 100 μg/mL SAE for 24 h. The expressions of p-EGFR, p-caveolin, P-Src, p-Akt, p53, Bcl-2, Bak, PARP, and cleaved PARP. Pro-caspase 3, cleaved-caspase 3, and β-actin were analyzed by Western blotting (each experiment, n=3). All western blotting images were quantitatively represented using ImageJ (National Institutes of Health). Statistical analyses were performed using t-tests; *P < 0.05, **P < 0.01, ***p < 0.001 compared to N groups (each experiment, n= 3). The error bars represent the standard error. N., Negative control; S., SAE; G., Gefitinib; L., LY294002.

To determine how EGFR and Akt affect the induction of anoikis in HCT116 colorectal cancer cells by SAE, we treated the cells with 20 μM EGFR inhibitor and 20 μM Akt inhibitor (gefitinib and LY294002) with or without SAE (100 μg/mL) for 24 h. WST-1 assays were subsequently performed. The cell viability were observed to be 64.8% with SAE treatment, 77.9% with gefitinib treatment, 73.6% with LY294002 treatment, 48.8% with the combined treatment of SAE and gefitinib, and 49.1% with the combined treatment of SAE and LY294002. EGFR and Akt were found to affect the growth and proliferation of HCT116 colorectal cancer cells, and SAE was also found to exhibit inhibitory effects on proliferation, similar to the inhibitors (**Figure 5A**). To investigate the tendency of anoikis induction by EGFR and Akt regulation, Western blotting was performed by treating HCT116 colorectal cancer cells with SAE, gefitinib, and LY294002 under the same conditions as the above experiment. Briefly, inhibition of EGFT and Akt would lead to decrease in p-EGFR, p-caveolin-1, p-Src, and p-Akt decreased; and as p53 increases, Bcl-2 would decrease and Bak would increase, while activation of caspase 3 would decrease pro-caspase 3 and increase cleaved-caspase 3, which would deactivate PARP, and cause an increase in cleaved-PARP. Our results showed that such expression level of each of these proteins were more prominent when treated in combination with SAE and each of the inhibitors than when treated with a single substance. Thus, it was confirmed that anoikis were induced through the regulation of the EGFR/Akt pathway of SAE by further inhibiting the EGFR/Akt pathway, which was not inhibited by the inhibitor (**Figure 5B**).

### *In Vivo* Inhibition of Tumor Growth by SAE

**Figure 6:**
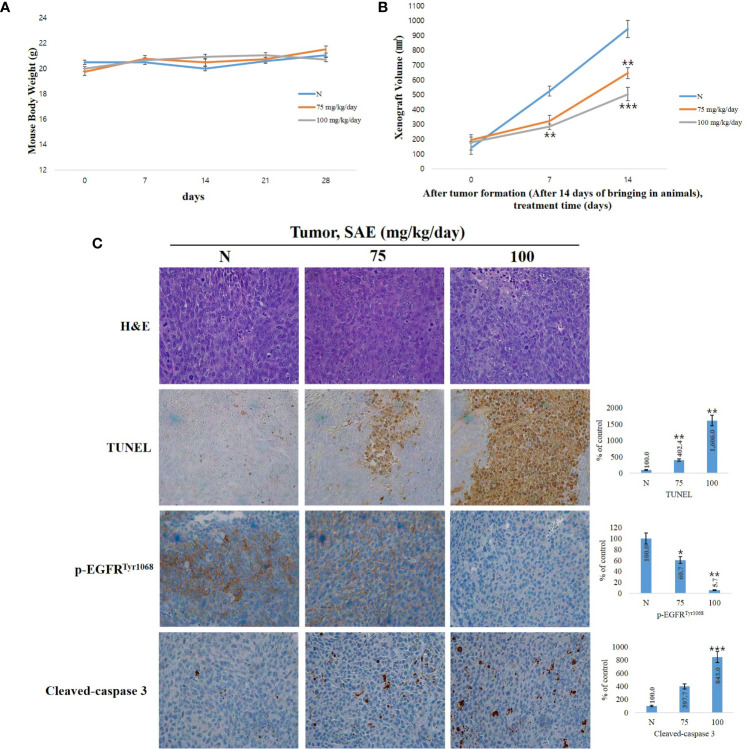
SAE suppresses tumor growth *in vivo*. **(A)** Measurement of body weight. **(B)** Measurement of tumor size. SAE treated groups (75 or 100) were compared against untreated groups (N). Statistical analyses were performed using t-tests; *p < 0.05, **p < 0.01, ***p < 0.001. N.S., not significant. **(C)** H&E staining, TUNEL assay and immunohistochemistry (IHC) of specific proteins (p-EGFR, cleaved-caspase 3). Tumor sections were observed using an optical microscope at ×400. All images were quantitatively represented using ImageJ (National Institutes of Health). Statistical analyses were performed using t-tests; *P<0.05, **P<0.01, ***p < 0.001 compared to N groups (each experiment, n= 3). The error bars represent the standard error. N, Negative control; 75, SAE 75 mg/kg/day; 100, SAE 100 mg/kg/day.

When anoikis, apoptosis due to loss of cell adhesion, occurs normally, it can partially inhibit tumor cell invasion, metastasis, and angiogenesis, and it is also involved in the inhibition of tumor proliferation (29). To investigate tumor suppression by SAE *in vivo*, we established a HCT116 colorectal cancer xenograft model by transplanting human HCT116 cells into 4-week-old male Balb/c-nu mice. We found no changes in body weight in both the control group and the SAE groups (75 mg/kg/day and 100 mg/kg/day in PBS) (**Figure 6A**); however, tumor size was reduced to a greater extent in the SAE groups than in the control group. In addition, tumor size decreased effectively as the concentration of SAE increased (**Figure 6B**). The number of TUNEL-positive cells represents a marker of apoptosis, as indicated by DNA degradation. To analyze apoptosis in all groups of tumor tissues, we performed a TUNEL assay and observed that TUNEL-positive cells increased in all SAE concentration groups compared with the control group, and a dose-dependent increase was confirmed (**Figure 6C**). p-EGFR and cleaved-caspase 3 expressions were further evaluated by immunohistochemistry. Compared with the control group, lower p-EGFR and higher cleaved-caspase 3 expression levels were demonstrated in all SAE concentration groups, which indicated a concentration-dependent effect of SAE on both factors (**Figure 6C**). These results demonstrated the tumor suppression effects of SAE and confirmed that SAE regulates the activity of EGFR and caspase 3 in tumor cells.

## Discussion

Recently, many cancer-related studies have been conducted on traditional medicinal plants, with the focus of identifying new compounds with physiological effects and investigating their potential value as anticancer drugs (30). These studies provide the basis for developing anticancer drugs that have fewer side effects and that can serve as an alternative to mitigate commercial anticancer drug resistance (31, 32). Since the characteristics of many cancer cells, such as invasiveness and metastasis, are mediated by anoikis resistance, targeting and treating cancer cells are often difficult. This thereby suggests that direct induction of anoikis that would cause a loss of adhesion in cancer cells can partially inhibit cancer cell proliferation, invasion, and metastasis (33). This study therefore investigated the mechanisms of anoikis induction and tumor suppression in HCT116 colorectal cancer cells following exposure to SAE, an extract of traditional medicinal plants.

Scaphium affine is also known as S. lychnophora. β-sitosterol is also found in seeds of S. lychnophora (Scaphium affine) which was discovered by Wang et al. (34). The anticancer effects of β-sitosterol has been shown in colorectal cancer cells (35, 36). Therefore, β-sitosterol was established as an indicator component in this study, and qualitative evaluation through HPLC was performed to determine whether the indicator component was extracted when extracting the SAE used in the study (37, 38). With the assumption that SAE contains other apoptosis-inducing components besides β-sitosterol, we are investigating the qualitative evaluation of other components that induce apoptosis in HCT116 colorectal cancer cells. Additionally, a quantitative evaluation is being conducted to confirm the effect of β-sitosterol.

The WST-1 assay showed that cytotoxicity was induced in HCT116 and HT29 colorectal cancer cells upon exposure to SAE. Dose-dependent cytotoxicity was observed in both cell lines, and greater cytotoxicity was observed in the HCT116 cell line. When the same method was applied to CCD841 normal colon epithelial cells, cytotoxicity was not observed at all concentrations. These results indicated that SAE cytotoxicity is specific to colorectal cancer cells.

Anoikis refers to apoptosis induced by abnormal adhesion and loss of adhesion. Anoikis are intrinsic apoptosis induced by blocking specific signals that regulate the interaction between cells and the extracellular matrix (ECM). Therefore, in order to confirm normal anoikis induction, it is necessary whether or not apoptosis is induced. Therefore, it was confirmed by Annexin V that SAE induces apoptosis in a dose-dependent manner in HCT116 colorectal cancer cells. Additionally, given that the normal functioning of anoikis inhibits the invasion and metastasis of cancer cells, a cell invasion assay was performed to investigate the inhibitory effects of SAE on cell invasion. Our findings confirmed that SAE dose-dependently inhibits the invasiveness of HCT116 colorectal cancer cells, and that these results are associated with the anoikis-inducing effects of SAE.

EGFR plays a key role in the induction of anoikis (39, 40). When EGFR is activated, caveolin-1, which inhibits anoikis and promotes tumor metastasis, is also activated, and Src, a signaling transducer, subsequently activates Akt to promote cancer cell and tumor proliferation (41). Activation of this cascade increases anoikis resistance; therefore, inhibition of both EGFR and Akt are required to induce anoikis. To examine whether anoikis is induced by SAE, we assessed factors associated with anoikis induction using Western blotting. It was observed that EGFR, caveolin-1, and Src were inhibited by SAE, which led to the loss of adhesion and the disruption of signal transduction, resulting in inactivation of Akts that are directly involved in cell growth and proliferation. Furthermore, the tumor suppressor p53 was observed to be increased, while Bcl-2, which maintains mitochondrial membrane potential, was found to be suppressed, and Bak, which increases mitochondrial membrane permeability, was increased. In addition, increased cleaved-caspase 3, an activated form of caspase 3, and decreased pro-caspase 3, an inactivated form, and fragmented PARP, which repairs DNA, were observed, which resulted in an increase in cleaved-PARP (26, 42–45). Accordingly, expression of the identified anoikis-inducing factors showed a tendency to increase or decrease with SAE in a dose-dependent manner.

To demonstrate anoikis induction through the EGFR/Akt signalling pathway, cell viability and changes in the trends of associating factors were assessed through treatment with gefitinib (EGFR inhibitor) and LY294002 (Akt inhibitor). Greater cytotoxicity was observed when cells were treated with a combination of SAE and EGFR/Akt inhibitors than with SAE or EGFR/Akt inhibitors alone in the WST-1 assay. The Western blot assay also showed that anoikis-related factors tend to show stronger effects when treated in combination than when treated with SAE or inhibitor alone. Due to the similar effects observed between SAE and EGFR/Akt inhibitors and stronger effects observed by treated in combination, we confirmed that SAE induces anoikis through the EGFR/Akt pathway.

To confirm the tumor suppressing effects of SAE, a xenograft model of HCT116 colorectal cancer cells was established, and we found no changes in body weight of the experimental animals in the SAE group, alongside reduced tumor size when compared to the control group. In addition, the TUNEL assay confirmed that SAE increased apoptosis rates in tumor cells and decreased p-EGFR and increased cleaved-caspase 3 in a dose-dependent manner.

In conclusion, this study showed that SAE containing physiological compounds such as β-sitosterol, which have been shown to have anticancer effects in colorectal cancer cells, induces anoikis through the EGFR/Akt pathway. This study is the first to report the *in vitro* and *in vivo* anticancer effects of SAE on HCT116 colorectal cancer cells. This study provides a basis for the potential use of SAE as an alternative anticancer drug for anoikis resistant-related invasion and metastasis. In addition, this study may facilitate separation and screening of compounds with anti-cancer potential for substitution of colorectal cancer chemotherapeutic drugs.

## Data Availability Statement

The original contributions presented in the study are included in the article/supplementary material. Further inquiries can be directed to the corresponding author.

## Ethics Statement

The animal study was reviewed and approved by Hannam University Animal Experimental Ethics Approval Committee.

## Author Contributions

HK, G-HN, MK, and S-YK carried out the WST-1 assays, flow cytometry (cell cycle arrest and Annexin V staining), and Western blotting, established the tumor xenograft model, and performed the H&E staining, TUNEL assay, and immunohistochemistry. HK and G-HN performed the HPLC analyses. HK and Y-MK wrote the manuscript. All authors contributed to the article and approved the submitted version.

## Funding

The authors declare that this study received funding from the Forbio Ltd. The funder was not involved in the study design, collection, analysis, interpretation of data, the writing of this article or the decision to submit it for publication.

## Conflict of Interest

The authors declare that the research was conducted in the absence of any commercial or financial relationships that could be construed as a potential conflict of interest.
